# Humor as a Reward Mechanism: Event-Related Potentials in the Healthy and Diseased Brain

**DOI:** 10.1371/journal.pone.0085978

**Published:** 2014-01-29

**Authors:** Armand Mensen, Rositsa Poryazova, Sophie Schwartz, Ramin Khatami

**Affiliations:** 1 Department of Sleep Medicine, Clinic Barmelweid, Barmelweid, Switzerland; 2 Department of Neurology, University Hospital Zurich, Zurich, Switzerland; 3 Department of Neuroscience, University of Geneva, Geneva, Switzerland; INSERM, France

## Abstract

Humor processing involves distinct processing stages including incongruity detection, emotional response, and engagement of mesolimbic reward regions. Dysfunctional reward processing and clinical symptoms in response to humor have been previously described in both hypocretin deficient narcolepsy-cataplexy (NC) and in idiopathic Parkinson disease (PD). For NC patients, humor is the strongest trigger for cataplexy, a transient loss of muscle tone, whereas dopamine-deficient PD-patients show blunted emotional responses to humor. To better understand the role of reward system and the various contributions of hypocretinergic and dopaminergic mechanisms to different stages of humor processing we examined the electrophysiological response to humorous and neutral pictures when given as reward feedback in PD, NC and healthy controls. Humor compared to neutral feedback demonstrated modulation of early ERP amplitudes likely corresponding to visual processing stages, with no group differences. At 270 ms post-feedback, conditions showed topographical and amplitudinal differences for frontal and left posterior electrodes, in that humor feedback was absent in PD patients but increased in NC patients. We suggest that this effect relates to a relatively early affective response, reminiscent of increased amygdala response reported in NC patients. Later ERP differences, corresponding to the late positive potential, revealed a lack of sustained activation in PD, likely due to altered dopamine regulation in reward structures in these patients. This research provides new insights into the temporal dynamics and underlying mechanisms of humor detection and appreciation in health and disease.

## Introduction

Research in the field of humor processing has taken several key steps over the past two decades, both in terms of its underlying neurobiology and its psychological functions [1], [2]. However, two major dimensions of humor processing have been left relatively unexplored to date. Firstly, neuroimaging studies have largely focused on the spatial characteristics of humor processing using functional magnetic resonance imaging (fMRI; [3]–[5]), while few studies have examined the temporal dynamics of these processes using magneto/electroencephalography (MEG/EEG). EEG and MEG studies to date have focused almost exclusively on the dynamics of verbal humor comprehension; with a particular focus on the so-called N400 component [6]–[8]. Only recently has visual humor been assessed using EEG in a study on emotional suppression [9]. This study focused on participant's active manipulation of the late positive potential (LPP), which has been linked to the underlying activity in reward related structures [10], [11]. Secondly, many fMRI studies found that regions implicated in humor appreciation and experiencing positive rewards are largely overlapping and include dopaminergic regions of the midbrain and ventral striatum, as well as the amygdala [3]–[5], yet no study has used humorous stimuli as a specific reward signal.

To closer examine the underlying mechanisms at each processing stage, in particular the engagement of the dopaminergic and hypocretinergic reward system, we tested humor as feedback stimuli in patients with Narcolepsy-Cataplexy (NC) and idiopathic Parkinson's disease (PD). These patient populations are of interest because of their striking clinical symptoms in response to humorous stimuli and well characterized deficits in reward processing. Specifically, humor and laughing are the strongest trigger for cataplexy, a sudden loss of muscle tone triggered by emotions and the clinical hallmark of the NC [12]. NC is caused by a deficit in the hypothalamic hypocretin system, which also has strong interactions with the reward system [13]–[15]. While the motor components of cataplexy have been extensively investigated and attributed to inhibition of spinal alpha motoneurons mediated by ponto-medullary activity, emotional processing itself and the mechanisms of how emotions compromises the control of motor system remains essentially unknown [16]. In previous fMRI studies of NC we identified dysfunctional activation patterns in midbrain and ventral striatal reward related circuits and an increased amygdala activation in response to humorous pictures [17], [18].

Using EEG, we aimed to compare the temporal dynamics and various stages of humor processing in NC and healthy controls with those of PD because of the latter groups well know impairments to the normal functioning of dopaminergic system and their blunted response to humor. PD is characterized by a progressive loss of dopamine producing neurons in the dorsal striatum; predominantly leading to a disturbance in motor functioning. However, as the disease progresses to ventral portions of the striatum, or as a result of treatment with dopaminergic agents, PD patients also show deficits in mesolimbic reward functions [19], [20], as well as blunted emotional responses and deficits in joke comprehension [21]. Despite these previously researched impairments to humor and reward processing on both the behavioral and neuroimaging levels, this study is the first to examine these issues using EEG in these populations. Based on our previous fMRI finding of enhanced activity in the amygdala and right inferior parietal cortex in NC during on humor processing we hypothesized that narcolepsy-cataplexy patients would show ERP differences in distinct stages of processing of humor. In particular we expected that these effects might elicit early increases in ERP amplitude during humor feedback related to a rapid emotional and attentional orienting response. Furthermore, we expected a reduction of the rewarding value of humor due to impaired dopaminergic activity in the PD group, which would result in a reduction of amplitude for later ERP components.

## Materials and Methods

### Participants

Nineteen NC patients, 15 PD patients and 19 healthy controls were recruited from the University Hospital Zurich and Clinic Barmelweid. The final statistical analysis of the ERP datasets was performed on 12 participants from each group after EEG and behavioral inclusion criteria was met (see analysis section). Table 1 provides the demographic information for the participants. HLA typing was positive for HLA-DQB1*0602 in all 11 NC patients tested (no data for one patient). Hypocretin in the CSF could be obtained in 8 of the 12 patients and all showed undetectable levels. International criteria was used in the diagnosis of Parkinson's disease [22]. Each participant signed an informed consent form prior to the start of the experiment. The study was independently approved by both the cantonal ethical commissions of Zurich and Aarau, Switzerland.

**Table 1:** Participant demographics and statistical differences.

	Healthy Controls	Narcolepsy Cataplexy	Parkinson'sDisease	Statistics
**Age**	34.1 (4.1)	40.2 (3.0)	68.1 (2.2)	F_2,33_ = 31.87p<0.001
**Gender**	6 Male6 Female	5 Male7 Female	7 Male5 Female	?^2^_36_ = 0.67p = 0.717
**ESS**	5.0 (1.0)	16.9 (1.4)	7.3 (1.2)	F_2,33_ = 30.23p<0.001
**BDI**	2.2 (0.7)	10.6 (2.5)	7.9 (1.0)	F_2,33_ = 6.99p = 0.003
**ULN**	5.3 (0.7)	24.5 (2.5)	6.9 (1.6)	F_2,33_ = 35.78p<0.001
**Medication**	None (12)	None (5)Sodium Oxybate (6)Modafinil (4)*	None (1)Levodopa (11)Clonazepam (3)+	

ESS = Epworth Sleepiness Score, BDI = Beck's Depression Inventory, ULN = Ullanlinna Narcolepsy Scale.

* 3 NC patients took both Sodium Oxybate and Modafinil.

+ 3 Parkinson's patients took a combination of Levodopa and Clonazepam.

As shown in Table 1 and expected from the distinct pathologies, NC and PD patient groups differed in their levels of sleepiness, with NC patients rating significantly higher on the Epworth Sleepiness Scale [23](ESS; a scale from 0 to 24 points indicative of long-term daytime sleepiness), depressive symptoms (measured using the Beck Depression Inventory), and ages, with PD patients being older than the other two groups. Given the inherent differences between patient groups, and our previous finding for NC, healthy controls were selected and matched for age and gender with respect to the NC group. Crucially, due to both ethical and clinical restrictions, 7 of the 12 NC patients maintained their regular level of medication during the experiment. Furthermore, 11 of the 12 PD patients kept to their normal dosages of medication, with one patient being drug-naive. These 11 patients were all taking medication which in different forms increases the amount of available dopamine (L-Dopa (Madopar®), Rotigotine (Neupro®), or Rasagiline (Azilect®)). Although the particular effects of continuing dopaminergic treatment in our PD patients are fairly complex and difficult to predict [24], maintaining patients medication reduced the likelihood of complete apathy in this patient group [25], and provides a more realistic everyday perspective as the vast majority of PD patients do indeed receive treatment. The inherent demographic and treatment differences between groups are further considered in the analysis and discussion section.

### Task

**Figure 1:**
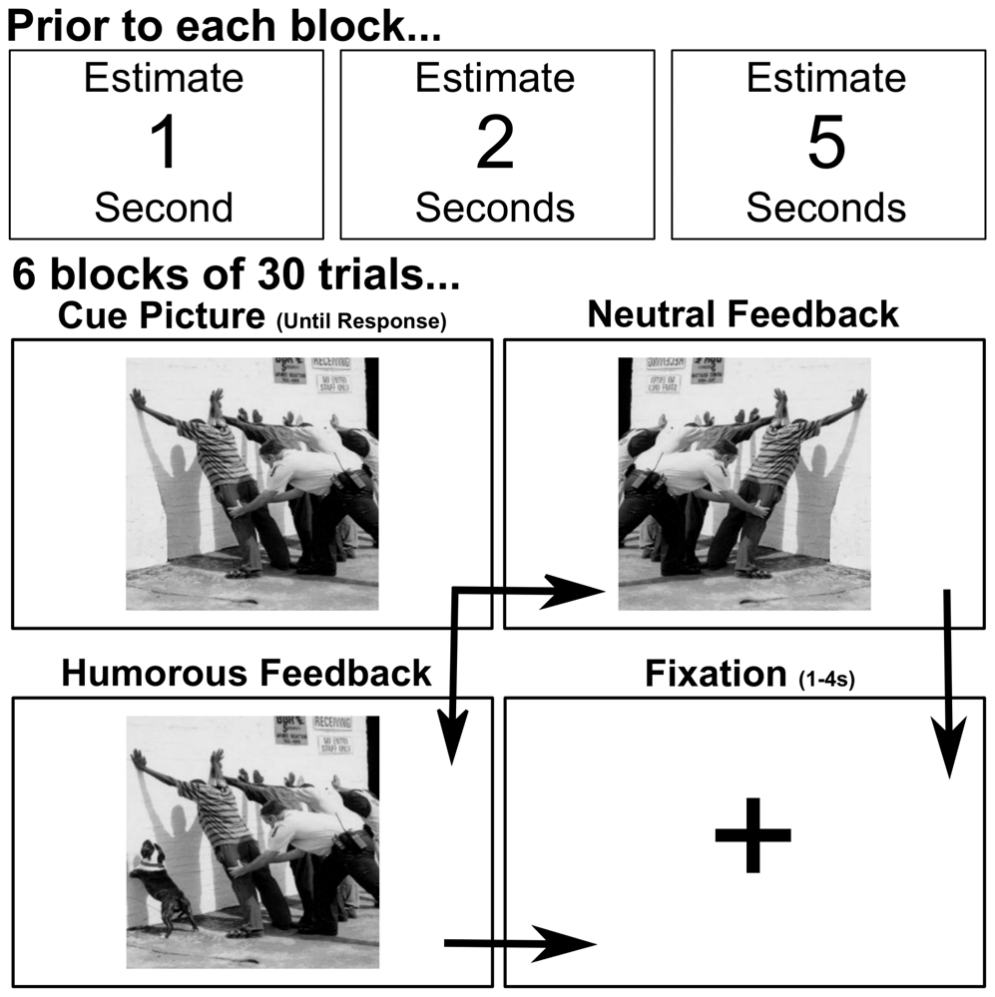
Experimental task. At the start of each block (of 30 trials), the participant was instructed to estimate 1, 2, or 5 seconds as soon as the first image was presented. 6 blocks in total. The first picture presented was always a neutral image, then depending on the accuracy of the participant's estimation either a positive, humorous picture was presented, or the initial neutral picture was horizontally flipped. A fixation cross was presented for a random duration between 1 and 4 seconds (mean 2.5+/−1) before the onset of the next trial.

Participants completed a time estimation task and then were given subsequent feedback based on their performance. The task was presented in a total of 6 blocks of 30 trials each. Prior to each block the participant was informed that they would be required to estimate durations of either 1, 2 or 5 seconds. Each trial consisted of a neutral picture presented as a cue indicating when they should start their estimation. Participants were instructed that once they believed the indicated duration of time had passed to that they should press a button with their index finger of their dominant hand. After approximately one second (randomly jittered), participants were presented with either a horizontally flipped version the same neutral image, or a slightly altered version of the image which made the picture a humorous one (see Figure 1). Each trial ended with a fixation cross lasting 1–4 seconds (normally distributed jitter around 2.5 seconds), leading directly to the next cue-picture. Participants were made aware that estimations within a certain window around the target time would result in changes to the picture to make it potentially humorous, whereas the image would simply be flipped if their response was outside this window. Importantly, the criteria for successful completion of the trial were constantly changed so that learning in this task is minimal.

Trial success or failure, and hence whether the humorous or neutral version of the picture was presented, was determined by whether the participant's estimate was within a certain +/− time window of the target. The window for success was initially set to 500 ms around the target and adjusted on a trial to trial basis with correct responses shortening this window by 33%, while incorrect responses would lengthen this window by 33%. This adjustment ensured that participants received approximately 50% successful feedback over the course of the experiment, and that feedback remained linked to their actual performance. Note that the time estimation task itself was unrelated to the humor experiment and it was not our intent to implement a learning algorithm. Thirty-six distinct images were selected as the funniest images (mean humor intensity of 2.2/3), from the database of 100 total images used in our previous study [17]. The order of images presented was pseudo-randomized in that the same image was never presented consecutively and all images were presented a total of 5 times.

### EEG Recording and Processing

EEG was recorded from 125 sites on the scalp using a HydroCel Geodesic Sensor Net by Electrical Geodesics, Inc. (EGI) [26], sampled at 1000 Hz. Impendences were kept below 30Ω on all channels. All EEG pre-processing was performed using BrainVision's Analyzer (version 2; Brain Products, Munich, Germany) and Matlab (MathWorks, Natick, MA). For all participants, bandpass filters were applied between 1 and 30 Hz using a modest 12/24 dB slope including a notch filter at 50 Hz to remove mains-power noise. Data was then down-sampled to 250 Hz, individual bad channels were removed after visual inspection (never more than 6 channels per participant), and classic independent component analysis was performed over the entire length of the continuous data in order to remove components of the EEG associated with artifacts (i.e. electrocardio/oculo/myography artifacts; rhythmic tremor related artifacts in the PD group). The activity in the missing channels was then estimated through topographical interpolation using 3D splines [27]. Channels were then re-referenced to the average electrical activity over all channels [28]. Semi-automatic criteria were used to determine the presence of any remaining spurious artifacts which were then marked and eliminated from segmentation on an individual channels basis (maximal allowed voltage step of 25 µV/ms; maximal absolute difference of 75 µV/ms over 200 ms window; EEG within −150 µV and 150 µV in amplitude). A mean of 5.0 (SD = 2.4) trials of the 180 total were removed for each participant using this criteria. ERPs were created using a baseline period of 200 ms prior to the picture presentation to 1000 ms after the event. For ERPs locked to the cue picture presentation, only the segments for the 2 s and 5 s estimation trials were used so as minimize any overlap of brain activity associated with the decision making and motor preparation required for the actual task response.

Current limitations in the analysis procedure as well as general guidelines in statistical analysis meant that the ANOVA required equal sized groups. Data from two PD participants could not be calculated due to technical artifacts in the recording leading to several missing blocks of data. A further PD patient was removed due uncorrectable motor artifacts by filtering or ICA leading to fewer than 30 trials in the final ERP waveform. Thus an upper limit of 12 participants for the two other groups was set by the PD population. Apart from technical problems in the EEG recording itself, a further 3 participants final ERP waveforms from the controls and NC group were not used in the final analysis and selected based on the highest standard deviation in the baseline period of 200 ms; an indicator for the overall quality of the ERP waveform resulting in 12 participant datasets for each group.

### EEG Analysis

Statistical analysis of the ERP dataset comparisons was performed using a threshold-free cluster-enhancement technique (TFCE), followed by maximum non-parametric permutation statistics for significance testing [29]. This robust statistical approach allows us to analyze all channel-sample pairs across participant groups and conditions while both controlling for multiple comparisons and maintaining optimal sensitivity to potential signal differences. In order to examine both group differences and the effect of condition together, an analysis-of-variance approach (TFCE_ANOVA_) was used as the initial statistic for further permutation analysis [30], [31]. Here, F-ratios for the main effects of group (NC, PD or healthy controls), and condition (neutral or humorous pictures), as well as their interaction effect are calculated for the original group datasets. The datasets are then randomly permuted across conditions first, then groups second (to ensure that a single participants condition files are never separated), and the F-ratios for this new dataset are calculated as well. The neighborhood of each channel-sample pair, both in terms of nearby channels and time points are then examined in order to calculate the amount of statistical support provided by its neighbors. Dependent on the amount of support (or not) each data-point has, its value is either enhanced or suppressed to give rise to new TFCE values which represent not only the statistical strength found specifically for that channel in the mass uni-variate approach, but also whether neighboring channels and time points show a similar pattern of activity (see [29], for complete details). This process was repeated with 10000 randomly permuted datasets to obtain an empirical distribution of TFCE values from which to determine statistical significance. Although the method principally relies on detecting local differences in amplitude, by enhancing these statistics using both information from neighboring channels and time points we are able to still detect smaller changes in amplitude reflective of larger shifts of peak location (topography differences), or time (latency shifts).

Two separate TFCE_ANOVA_ analyses were carried out: a one-way TFCE_ANOVA_ examined the presentation of the cue-picture across the three experimental groups; the second, a three-by-two mixed-factor TFCE_ANOVA_ on the feedback picture presentation examined the group effect for both the neutral and humorous pictures. As with standard ANOVA analyses, separate post-hoc analyses using independent t-tests as the initial statistics followed by TFCE and permutation statistics (TFCE_T_), were used when appropriate to determine which of the three groups differed from one another. Finally, a single independent TFCE_T_ was performed to compare the medicated NC patients against the non-medicated NC patients for potential effects of treatment differences on humor processing. Since PD patients maintained their levels of medication, clinically adjusted to their specific motor symptoms, such an analysis is not possible for this group of patients.

Statistical alpha thresholds of interest were set at 0.05 for main effects and 0.20 for interaction effects. This low threshold for interactions was chosen because permutation of raw data has been shown to be particularly weak in the detection of interaction effects [30]. Any interaction of interest was then subjected to more classical analysis-of-covariance (ANCOVA), in order to confirm or reject the initial finding, as well as to allow for the inclusion of covariates into the model. This then allowed us to assess whether participant's age or sleepiness may have explained any ERP differences. Since both ESS and age are inherently linked to the group differences, new constructs of each were created by subtracting away the group means [32], [33]. This essentially leaves the individual variation of each measure within the group intact but statistically makes the constructs mathematically orthogonal with respect to the group differences as not to violate the basic rule of independent predictor variables in the ANCOVA. Depression scores significantly correlated with a participants ESS and were thus left out of this analysis (*r*_35_ = 0.33, p = 0.047). In addition main effects found were subject to additional post-hoc testing for the significant regions of interest while also including ESS, age as covariates. Additionally for the later differences, the amplitude values of early components were also included in the model to investigate whether early differences were predictive of late ERPs.

## Results

### Cue-Picture Presentation

**Figure 2:**
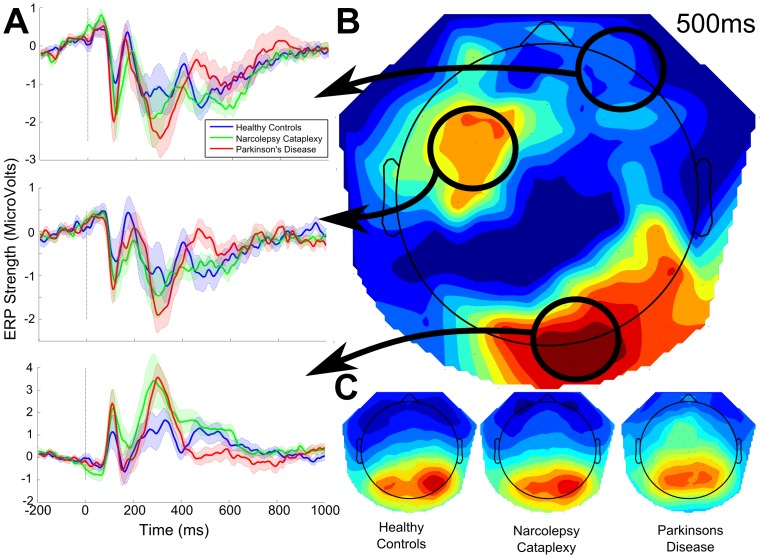
Event-related potentials (ERP) as well as the individual and statistical topographical maps following the presentation of the initial cue picture. Statistical analysis yielded no significant channels or time points for the duration of the ERP. A. Averaged waveforms from three distinct regions of interest for each experimental group. Colored shaded area indicates the standard error for each sample for each group. B. Topography of statistical differences at 500

The one-way TFCE_ANOVA_ with group as the main factor found no significant channel time pairs at the 0.05 level. Non-significant differences showed two late peaks at 480 ms and 600 ms over channels E75 (central-posterior; F_2,33_ = 5.174, p = 0.341) and E37 (left-central; F_2,33_ = 13.822, p = 0.219), respectively. Both peaks reflected higher ERP amplitudes for patient groups with respect to the healthy control participants. Figure 2 summarizes these non-significant findings.

### Feedback Presentation: Group Differences

**Figure 3:**
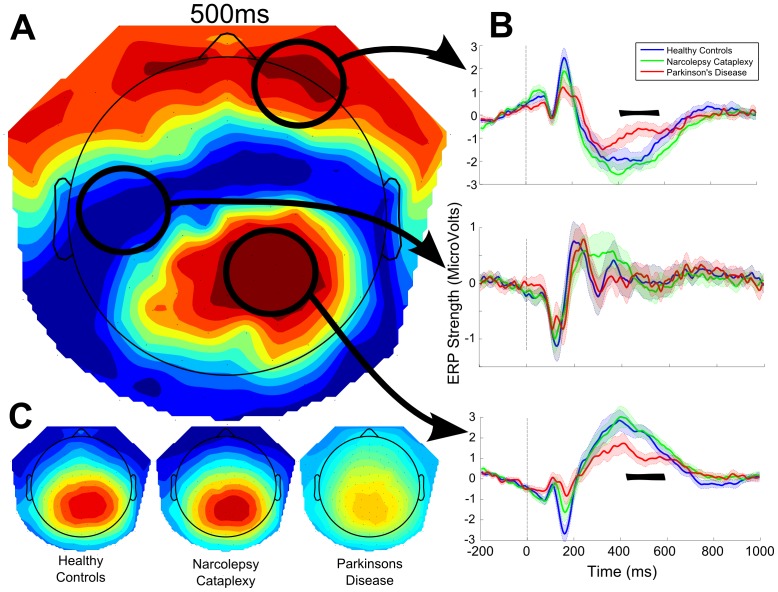
Event-related potentials (ERP) as well as the individual and statistical topographical maps for the maximum significance of group effect following feedback presentation. Analysis indicated a significant main group effect ranging from 460-posterior channels. A. Topography of TFCE statistics for the main effect of group at 500 ms Red areas indicate higher statistical differences. B. ERP waveforms from three distinct regions of the scalp for the entire time range of the ERP. Black bars over the ERP indicate significant time points for this channel whereas yellow and red areas in the TFCE_F_ topography indicate significant channels. C. Individual topographical maps below indicate similar ERP topographies for all groups at 500 ms, however the Parkinson's group showed significantly lower amplitudes.

The three-by-two TFCE_ANOVA_ showed several time points of significance at the 0.05 level. The main effect of group revealed a single cluster of significant channel-sample pairs (Figure 3). This cluster involved 31 distinct electrodes and ranged from 460 ms to 550 ms after feedback presentation. Group differences peaked at E86 (right-parietal) at 504 ms (F_2,33_ = 11.450, p = 0.026).

Post hoc analysis was performed using a single ANCOVA analysis with the orthogonal constructs of age, ESS, as well as two regions of interest for earlier components around 110 and 170 ms were used as covariates to examine the effect of group on a region of interest effectively describing the significant cluster indicated by the TFCE_ANOVA_ test (channels E78, E79, E85, E86, E87, E92, E93; from 488 to 512 ms). This test revealed that although a stronger earlier component at 170 ms significantly predicted stronger later amplitudes (F_1,29_ = 7.152, p = 0.012), the late potential was still primarily dependent on the participant's group (F_2,29_ = 7.826, p = 0.002). No other covariate reached significant levels. Planned contrasts to the ANCOVA revealed that the PD group was significantly different to both healthy controls (p = 0.008), and NC patients (0.001), with no group differences between controls and NC here (p = 0.499).

### Feedback Presentation: Condition Differences

**Figure 4:**
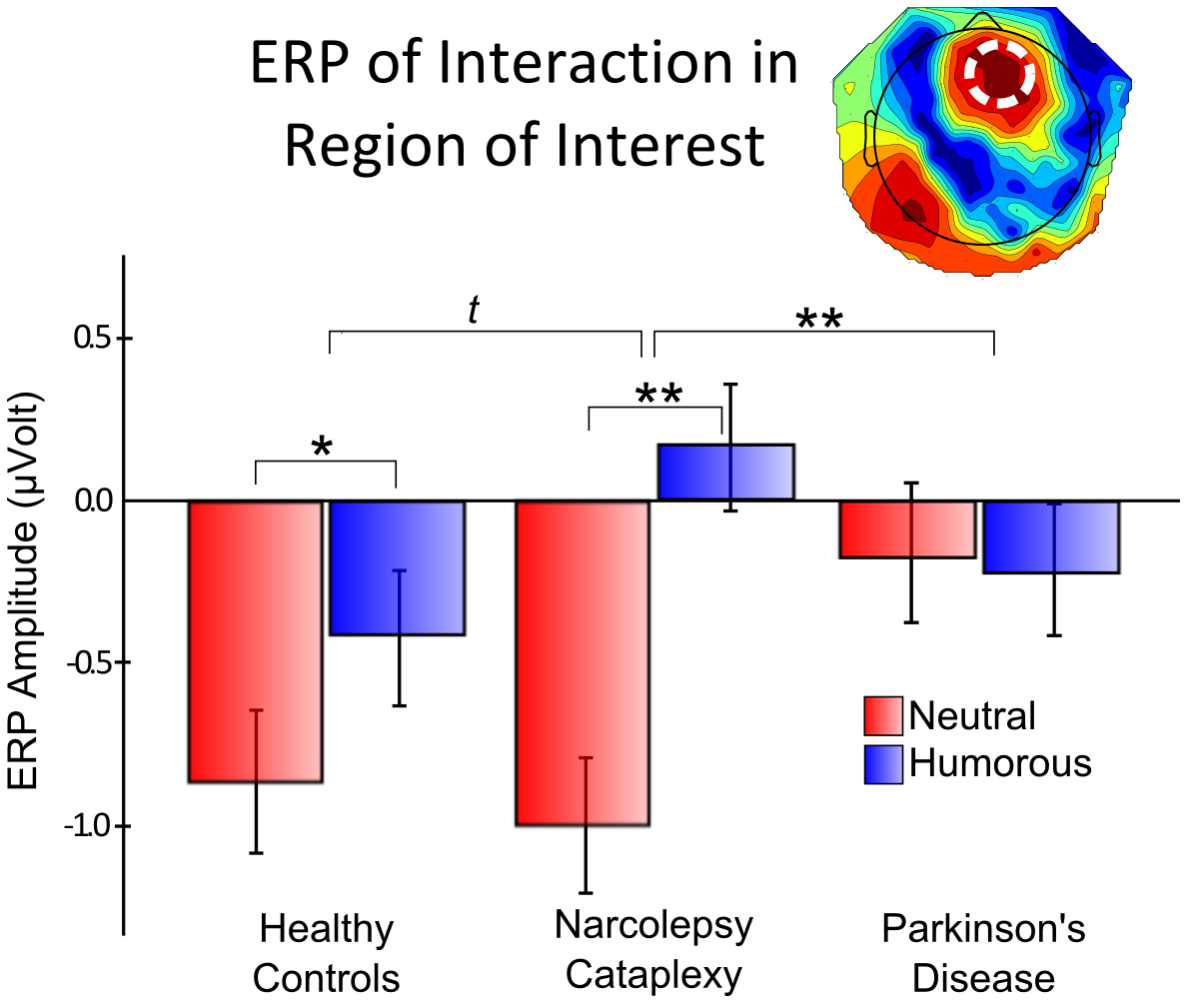
Event-related potentials (ERP) as well as the individual and statistical topographical maps for three maximum significance points of condition effects. Top section shows the early condition differences (peaking at 110 ms and 170 ms), in a representative channel over posterior electrodes. Middle section show the later conditional differences with variations in both topography and amplitude of the ERPs. Neutral pictures provoke left-posterior peaks while humorous feedback induces a fronto-central peak topography. Statistical differences are indicative of these peaks. Lowest bar shows the conditional effect of the late positive potential. Black bars over the ERP indicate significant time points for this channel whereas yellow and red areas in the TFCE_F_ topography indicate significant channels.

Figure 4 summarizes the main differences found between conditions, that is, whether a neutral or a humorous picture was given as reward feedback. Although the two ERPs began differentiating themselves from baseline levels as early as 20 ms post-presentation, the first significant cluster of differences peaked over channel E76 (central-posterior) at 112 ms (F_1,33_ = 80.900, p<0.001), reflecting a higher positive ERP amplitude for neutral pictures. Shortly thereafter around 170 ms, humorous pictures showed a higher overall negative amplitude peaking over channel E66 (left-posterior, F_1,33_ = 54.660, p<0.001). From 190 ms to 210 ms both conditions show a generally similar pattern of activity except for a small cluster of significant channels-sample pairs over the left posterior-temporal region, where humorous pictures have a significantly stronger negative amplitude (F_1,33_ = 10.339, p = 0.018).

After 240 ms, the main topographies of both conditions began differentiating in that neutral pictures generated a more left-posterior localized ERP, while humorous pictures generated a centralized ERP. Statistically this resulted into two main clusters of differences at the peaks of each ERP; E58 at 270 ms (F_1,33_ = 31.835, p = 0.002); and E105 at 280 ms (F_1,33_ = 19.140, p = 0.003) respectively. Around the same time a slower right-central positive ERP component was also found to show significant differences between 280 ms to 400 ms on the increasing initial slope of the ERP. The differences between the two conditions peaked over channel E93 at 360 ms (F_1,33_ = 31.592, p = 0.002). Here humorous pictures showed both a larger positive amplitude as well as shorter latency within the same topography. The negative reflection of this late positive potential was also found to be highly significant around the left fronto-temporal electrodes from 320 ms to 420 ms post-feedback (peak at E48, F_1,33_ = 43.943, p = 0.001). Finally, the two ERPs differentiated once again with the humorous condition showing sustained higher amplitudes whereas the neutral condition had returned to baseline levels. This effect started around 630 ms until 770 ms with a positive peak over channel E96 (right-posterior), and a negative peak over E11 (fronto-central), around 670 ms.

### Feedback Presentation: Group Condition Interaction

**Figure 5:**
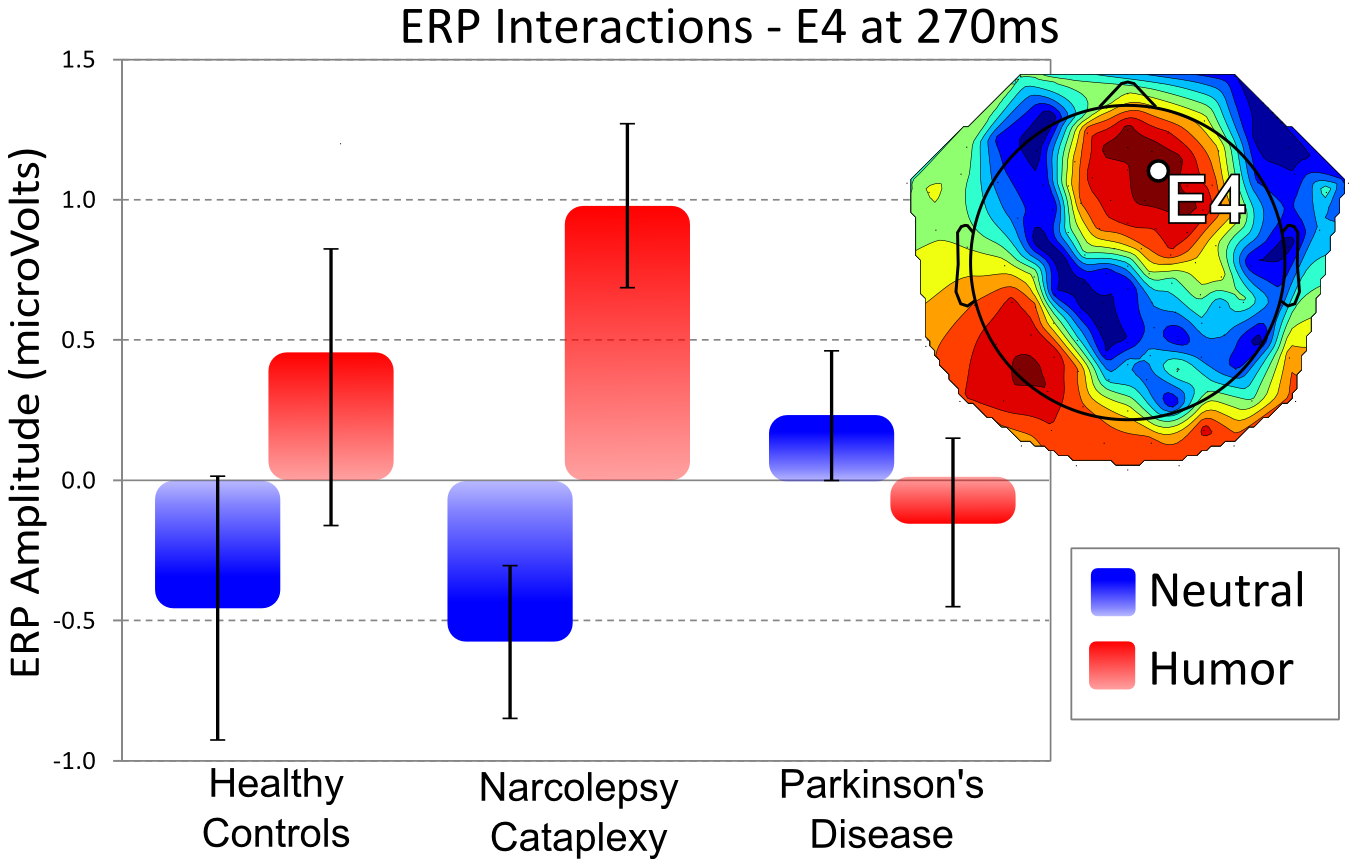
Individual event-related potential (ERP) amplitudes for each group and condition at peak interaction. Average amplitudes are shown for a right cento-frontal region of interest at 270 ms post-feedback presentation. For patients with Narcolepsy-Cataplexy and healthy controls humorous feedback produce significantly higher ERP amplitudes compared to neutral feedback. Parkinson's patients do not show this ERP pattern. Moreover, Narcolepsy-Cataplexy patients tended to show an even higher difference between the two feedback conditions than healthy controls. The imbedded topography indicates the region of interest as well as the statistical differences. Red values indicate more reliable statistical differences for the interaction between group and condition.

Group and condition interaction peaked over channel E4 (right-fronto-central), around 270 ms post-feedback (F_2,33_ = 11.631, p = 0.158; Figure 5). The fact that permutation of raw data is known to be insensitive in the detection of interaction effects [30], as well as the consistent topography of this interaction between 240 ms and 280 ms, calls for its further investigation. We thus submitted the difference ERP between the neutral and humorous feedback for a region of interest around the peak interaction channel-time point (channels E3, E4, E5, E10, E11, E118, and E124 from 264 ms to 276 ms), to a separate ANCOVA including age, ESS, as well as the two earlier components to examine whether the later differences were related to the earlier condition differences. We found that, only the main effect of group significantly accounted for the difference between the two conditions (F_2,29_ = 5.146, p = 0.012). The earlier ERP component at 170 ms showed trend levels (F_1,29_ = 3.194, p = 0.084), while all other covariates were not significant. Planned contrasts indicated the interaction was primarily driven by the divergence of values for the NC group in that maximal differences were found between the NC and PD group (p = 0.004), then NC and the healthy controls (p = 0.073), while the healthy controls and PD patients showed overall similar condition differences (p = 0.267). Moreover, when examining the effect of condition on each group separately, only the healthy controls (p = 0.036), and the NC patients (p = 0.004), showed significant differences.

### Medicated vs Unmedicated Narcolepsy-Cataplexy

An independent TFCE_T_ test reported no significant differences between medicated and unmedicated patients. However, with only five patients tested against seven, the power would clearly have been too low to expect for such a conservative approach to yield significant results. We therefore examined the maximal significance which peaked at E23 around 155 ms; T_10_ = 4.391, p = 0.492). The topography around this peak corresponded well to the main effect of condition around 170 ms described earlier. However, while the condition effect found between neutral and humorous pictures relied on amplitude differences, the main difference between medicated and unmedicated patients was clearly a latency shift. Perhaps somewhat counterintuitive is that the medicated patients showed a delayed time of onset and peak of this ERP by a mean of 32 ms (negative peak NC_med_ = 152 ms, NC_unmed_ = 184 ms). Importantly, this split group did not show ERP differences over E4 at 270 ms (the point of significant group and condition interactions), with mean differences of only 0.16 µV (uncorrected T-test; T_10_ = 0.258, p = 0.802).

## Discussion

Here we used high-density EEG to assess the temporal and topographical dynamics of humor processing as a reward signal. We included healthy participants and two further clinical groups of interest since NC patients are known to have abnormal emotional response to humor, and PD patients show impaired humor appreciation and reward processing. We were especially interested in humor processing in narcolepsy patients since humor is the main trigger of cataplexy, indicating a strong interaction of emotions and the motor system in NC. While our previous studies identified recruitment of amygdalae-hypothalamic and frontal areas during humor processing our findings now indicate that distinct stages of humor processing itself may contribute to mechanisms underlying cataplexy. The ERP results here suggest that the processing of humorous pictures may involve rapid differences in early processes followed by an emotional response to stimulus incongruity, then by a humor appreciation phase, during which the positive reinforcement value of the stimulus is processed. The later ERP component differences found in our patient groups provide important clues to the origins and functions of these components. The ERP at 270 ms found an increased response to humorous pictures in NC patients at 270 ms, compared to PD and healthy controls, while PD patients showed a late overall reduction in response amplitude to both neutral and humorous feedback after 500 ms. Given that the earlier components are not predictive of the later ERP differences, and thus are likely to be caused by distinct underlying mechanisms, each is discussed separately.

### Early evoked responses

The earliest ERPs generated by feedback presentation were found at 110 ms and 170 ms with larger positive amplitudes for neutral pictures and later larger negative amplitudes for humorous pictures respectively. Given that for both ERPs, differences were maximal over central posterior channels we hypothesized that both of these peaks correspond to visual processes. In terms of latency, magnitude, and topography, these ERPs correspond well to the well-researched visual evoked potentials P100 and N170. Previous research has found that low-frequency spatial characteristics (global) processing primarily occurs at the P100 mark while high-frequency spatial characteristics, fine-feature processing occurs at the N170 mark [34]–[36]. These theories may also best explain the differences found here since neutral pictures were created through a global transformation of the cue-picture (horizontal flip), and hence a higher P100 amplitude, while humorous pictures generally entailed a smaller, local addition to the cue-picture, hence resulting in the higher N170 amplitudes found. This may also explain the rapid shift of topography at the 200 ms mark, with its central positivity probably representing the less understood P2 ERP. This component is thought to handle more advanced processing of stimuli, such as feature detection of salient stimuli [37], and a further attention-lock on a relevant stimulus [38]. Although speculative, the fact that for the P2 we only found significantly stronger amplitudes for humorous stimuli in electrodes over the left temporal-occipital junction may reflect the typically higher BOLD activity commonly found in this area [3], [4], [39].

### Later Differences in Response to Humor feedback

The significant differences found around 270 ms have two important aspects. The first is that here topographic changes between the two conditions emerged, as opposed to the amplitude differences involved in earlier components, thus suggesting a divergence in the brain areas involved in processing. Moreover, the first group differences also appeared at this stage with overall reduced amplitudes in PD patients while NC patients tended to show specifically increased ERP amplitudes to humorous feedback. We interpret the increased response in NC patients as an increased sensitivity to humor indicating that dysfunctions in emotional processing at a relative early stage may contribute to the pathophysiology of cataplexy. The brain's increased sensitivity to humor may represent the initial step in triggering downstream processes that lead to an affective loss of muscle tone control. While downstream pathways of cataplexy have been extensively investigated and attributed to descending ponto-medullo-spinal activity similar to those underlying REM-sleep atonia, the mechanism of how humor induces motor weakness remains essentially unknown. Given that early ERPs are similar between the groups and differences emerge later at 270 ms we conclude that attentional or cognitive processes such as ambiguity resolution or appreciation of humor, but not initial visual processing, are critically implicated. The observed trend for increased ERP amplitudes for NC patients precludes attentional resources to explain the ERP differences that have been found in other studies with reduced amplitudes in these patients for a variety of tasks [40]–[42]. Since we used humor to activate reward system including the ventral striatum and amygdalae it is likely that increased sensitivity to humor is related to reward processing itself. The increase in NC may be the electrophysiological counterpart of our previous finding using fMRI which found a clear hyperactivity of the amygdala in these patients in response to humorous stimuli [17]. This raises two important possibilities in relation to patient's cataplexy, is this increased activity a reflection of an oversensitive amygdala which in turns acts on the motor system [43], [44]; or might it be an active, voluntary, and possibly learned suppression of emotional response in NC in order to avoid a cataplectic attack [45]. PD patients reduced amplitude here are also in line with this ERP reflecting amygdala activity in that these patients have shown structural [46], [47] and functional brain abnormalities [48], [49], as well as changes in behavior where PD patients are impaired on tasks known to involve the amygdala such as the Iowa Gambling Task and Game of Dice Task [50], [51].

The right central-positive ERP (280–650 ms) initially differed by main condition effects with humorous stimuli showing an earlier initial slope with higher amplitudes, and then again later when PD patients ultimately show reduced amplitude in line with an inability to sustain activation of the ERP. The properties of this ERP fit well with the LPP, primarily found in research on affective picture and reward processing [52]. This ERP generally consists of a large positive deflection over central electrodes between 300 and 600 ms, and has been reported to be more lateralized to the right hemisphere, as was also found in this research [53], [54]. This potential has been shown to be reduced when examining neutral pictures in comparison to those with emotionally salient stimuli and LPP amplitude has been shown to be positively correlated with the fMRI signal in mesolimbic reward structures for pleasant pictures [10], [11]. Hence, the earlier effect of condition likely reflects a faster and stronger association of the humorous pictures as a more emotionally salient reward; whereas the delayed response for neutral pictures may reflect the fact that although in and of itself emotionally neutral, it nonetheless represents a negative feedback to the reward system. In this framework, the finding that the ERP amplitudes of PD patients returns to baseline levels faster than either NC patients or controls may reflect their general DA dysregulation in structures of the reward system [19], [20], [55], especially for those patients on dopaminergic medication as most are [24], [56].

### Limitations

NC and PD patients and healthy controls differed on several clinical aspects, beyond alterations in dopamine and hypocretin systems. As expected, NC and PD patients scored higher for chronic sleepiness than healthy controls, with NC patients' sleepiness ratings still higher than those of PD patients. However, it is unlikely that sleepiness explains group differences in these data because maximum differences were observed between both patients groups (whereas the sleepiness pattern would predict strongest difference when comparing the patients to the control group). Furthermore, PD patients were significantly older than both NC patients and controls, but two lines of reasoning argue against age as a direct cause for the significant late ERP differences shown by PD patients. Firstly, we found no such significant differences in ERP for the presentation of the cue-picture, with NC and PD patients actually showing higher, albeit non-significant, overall amplitudes compared to controls for the late ERP component. Secondly, when within-group age variation was included as a covariate in post-hoc analyses it was shown to have no significant independent effect on the ERP amplitudes.

A second limitation of the present study relates to the potential influence of DA modifying medication in both patient groups. Although 5 of the NC patients were drug-free, 4 patients regularly took modafinil, and 6 were under sodium oxybate (3 patients were taking both medications). Modafinil is thought to increase the availability of extra-cellular DA levels by inhibiting DA transporters [57]–[59], while sodium oxybate primarily acts on the GABAergic system, but may also lead to the increase of DA levels in mesolimbic reward structures through the downstream disinhibition of DA neurons [60]. However, when comparing medicated and non-medicated NC patients, we found similar ERP amplitudes between both subgroups at the peak channel and time point of the interaction. It therefore seems unlikely that medication in the NC group played a major role in the results or their interpretation here. In the PD group, patients maintained their prescribed levels of medication. Although it is clear that under the effects of medication, the amount of available extracellular DA is bound to increase compared to baseline, it is nonetheless difficult to predict whether medication was sufficient for normal functioning of the mesolimbic reward areas, or in fact created a detrimental excess of DA [61], [62]. DA levels and effect post-medication have been shown to depend on baseline levels of DA in different portions of the ventral and dorsal striatum [63], disease progression [24], genetic variations [64], and the cognitive process under evaluation [65]. Further studies should examine drug-naïve PD patients as well as those on and off dopaminergic treatment in order to determine whether the lack of a sustained ERP was indeed due to dysregulation of their DA system, or perhaps even a desensitization of the reward system induced by prolonged DA treatment [24].
